# Supplementary material for: Modeling glioblastoma heterogeneity as a dynamic network of cell states
Source: Mol Syst Biol. 2021 Sep 16;17(9):e10105. doi: 10.15252/msb.202010105 (PMC8444284; doi:10.15252/msb.202010105)
Supplement: Supplementary file 6 — Source Data for Figure 5 [file MSB-17-e10105-s004.zip › Figure5A_sourcedata/GSEA_3017/nosigdb_stateE.GseaPreranked.1622795031093/RICHARDS_NOWAKOWSKI_SCIENCE_2017_MGE_DIV_UPREG.html]

Details for gene set RICHARDS\_NOWAKOWSKI\_SCIENCE\_2017\_MGE\_DIV\_UPREG[GSEA]

|  || Dataset | state63017 |
| Phenotype | NoPhenotypeAvailable |
| Upregulated in class | na\_neg |
| GeneSet | RICHARDS\_NOWAKOWSKI\_SCIENCE\_2017\_MGE\_DIV\_UPREG |
| Enrichment Score (ES) | -0.627828 |
| Normalized Enrichment Score (NES) | -3.7648933 |
| Nominal p-value | 0.0 |
| FDR q-value | 0.0 |
| FWER p-Value | 0.0 |
Table: GSEA Results Summary

  

Fig 1: Enrichment plot: RICHARDS\_NOWAKOWSKI\_SCIENCE\_2017\_MGE\_DIV\_UPREG      
 Profile of the Running ES Score & Positions of GeneSet Members on the Rank Ordered List

  

| PROBE | GENE SYMBOL | GENE\_TITLE | RANK IN GENE LIST | RANK METRIC SCORE | RUNNING ES | CORE ENRICHMENT || 1 | HSPA5 |  |  | 73 | 0.449 | -0.1150 | No |
| 2 | TMSB4X |  |  | 93 | 0.412 | -0.1433 | No |
| 3 | CALM2 |  |  | 191 | 0.303 | -0.2978 | No |
| 4 | CDK6 |  |  | 238 | 0.269 | -0.3703 | No |
| 5 | PFN2 |  |  | 265 | 0.253 | -0.4108 | No |
| 6 | SUPT16H |  |  | 276 | -0.251 | -0.4254 | No |
| 7 | H2AFY |  |  | 297 | -0.257 | -0.4562 | No |
| 8 | RAN |  |  | 298 | -0.257 | -0.4547 | No |
| 9 | YEATS4 |  |  | 300 | -0.258 | -0.4549 | No |
| 10 | AHI1 |  |  | 310 | -0.261 | -0.4679 | No |
| 11 | HMGA1 |  |  | 313 | -0.261 | -0.4696 | No |
| 12 | HNRNPUL2 |  |  | 314 | -0.262 | -0.4682 | No |
| 13 | PSIP1 |  |  | 319 | -0.263 | -0.4731 | No |
| 14 | HNRNPDL |  |  | 322 | -0.263 | -0.4748 | No |
| 15 | TUBG1 |  |  | 323 | -0.263 | -0.4734 | No |
| 16 | CNTLN |  |  | 326 | -0.264 | -0.4751 | No |
| 17 | RAD51C |  |  | 333 | -0.264 | -0.4833 | No |
| 18 | HNRNPH1 |  |  | 334 | -0.265 | -0.4818 | No |
| 19 | PAICS |  |  | 336 | -0.265 | -0.4819 | No |
| 20 | MCM5 |  |  | 337 | -0.265 | -0.4804 | No |
| 21 | RCC2 |  |  | 340 | -0.266 | -0.4821 | No |
| 22 | BRD8 |  |  | 341 | -0.266 | -0.4806 | No |
| 23 | KNTC1 |  |  | 352 | -0.269 | -0.4952 | No |
| 24 | NUDT15 |  |  | 356 | -0.270 | -0.4985 | No |
| 25 | PHF10 |  |  | 359 | -0.271 | -0.5002 | No |
| 26 | STAG1 |  |  | 360 | -0.271 | -0.4986 | No |
| 27 | ERI2 |  |  | 364 | -0.272 | -0.5019 | No |
| 28 | DLEU2 |  |  | 365 | -0.272 | -0.5004 | No |
| 29 | TMEM237 |  |  | 383 | -0.276 | -0.5262 | No |
| 30 | SLBP |  |  | 386 | -0.277 | -0.5278 | No |
| 31 | HNRNPA2B1 |  |  | 391 | -0.279 | -0.5327 | No |
| 32 | NFATC2IP |  |  | 397 | -0.283 | -0.5391 | No |
| 33 | SNRPG |  |  | 398 | -0.283 | -0.5375 | No |
| 34 | PPP2R3C |  |  | 400 | -0.283 | -0.5376 | No |
| 35 | DERA |  |  | 402 | -0.284 | -0.5376 | No |
| 36 | NASP |  |  | 410 | -0.285 | -0.5472 | No |
| 37 | SIVA1 |  |  | 412 | -0.286 | -0.5472 | No |
| 38 | MAGI1 |  |  | 413 | -0.287 | -0.5456 | No |
| 39 | ZNF273 |  |  | 415 | -0.288 | -0.5456 | No |
| 40 | CDK2 |  |  | 422 | -0.290 | -0.5536 | No |
| 41 | LINC00342 |  |  | 425 | -0.291 | -0.5552 | No |
| 42 | SEPT10 |  |  | 431 | -0.293 | -0.5616 | No |
| 43 | MYBL1 |  |  | 432 | -0.294 | -0.5599 | No |
| 44 | FAM76B |  |  | 434 | -0.294 | -0.5598 | No |
| 45 | CHRAC1 |  |  | 435 | -0.294 | -0.5582 | No |
| 46 | ZNF738 |  |  | 437 | -0.295 | -0.5581 | No |
| 47 | HMGXB4 |  |  | 438 | -0.296 | -0.5564 | No |
| 48 | TTL |  |  | 440 | -0.297 | -0.5564 | No |
| 49 | ZNF43 |  |  | 449 | -0.299 | -0.5676 | No |
| 50 | CEP57L1 |  |  | 450 | -0.299 | -0.5659 | No |
| 51 | CDC7 |  |  | 451 | -0.299 | -0.5642 | No |
| 52 | ACYP1 |  |  | 454 | -0.301 | -0.5657 | No |
| 53 | HNRNPD |  |  | 456 | -0.301 | -0.5656 | No |
| 54 | MCM4 |  |  | 457 | -0.301 | -0.5639 | No |
| 55 | SNRPE |  |  | 458 | -0.302 | -0.5622 | No |
| 56 | UBR7 |  |  | 459 | -0.302 | -0.5605 | No |
| 57 | CNTRL |  |  | 462 | -0.304 | -0.5620 | No |
| 58 | UPF2 |  |  | 465 | -0.304 | -0.5635 | No |
| 59 | DNMT1 |  |  | 466 | -0.304 | -0.5618 | No |
| 60 | BCHE |  |  | 471 | -0.305 | -0.5665 | No |
| 61 | SMC1A |  |  | 472 | -0.307 | -0.5647 | No |
| 62 | POLR3K |  |  | 474 | -0.307 | -0.5646 | No |
| 63 | EXOSC9 |  |  | 481 | -0.308 | -0.5725 | No |
| 64 | NOP56 |  |  | 483 | -0.309 | -0.5724 | No |
| 65 | NEDD1 |  |  | 491 | -0.311 | -0.5819 | No |
| 66 | ILF2 |  |  | 497 | -0.313 | -0.5882 | No |
| 67 | RFC2 |  |  | 498 | -0.313 | -0.5864 | No |
| 68 | PA2G4 |  |  | 501 | -0.314 | -0.5879 | No |
| 69 | GGH |  |  | 504 | -0.314 | -0.5893 | No |
| 70 | HNRNPH3 |  |  | 507 | -0.316 | -0.5907 | No |
| 71 | BARD1 |  |  | 510 | -0.318 | -0.5922 | No |
| 72 | DNA2 |  |  | 513 | -0.318 | -0.5936 | No |
| 73 | CCDC150 |  |  | 516 | -0.319 | -0.5950 | No |
| 74 | HAUS1 |  |  | 518 | -0.320 | -0.5948 | No |
| 75 | SUZ12 |  |  | 524 | -0.322 | -0.6010 | No |
| 76 | NUP62 |  |  | 529 | -0.323 | -0.6057 | No |
| 77 | LSM3 |  |  | 530 | -0.323 | -0.6038 | No |
| 78 | CEP192 |  |  | 531 | -0.324 | -0.6020 | No |
| 79 | CEP135 |  |  | 532 | -0.325 | -0.6002 | No |
| 80 | RFC5 |  |  | 534 | -0.326 | -0.5999 | No |
| 81 | PRIM1 |  |  | 539 | -0.328 | -0.6045 | No |
| 82 | H3F3A |  |  | 542 | -0.329 | -0.6059 | No |
| 83 | JADE1 |  |  | 543 | -0.329 | -0.6040 | No |
| 84 | MSH2 |  |  | 548 | -0.332 | -0.6086 | No |
| 85 | CDC25C |  |  | 549 | -0.332 | -0.6067 | No |
| 86 | GPSM2 |  |  | 550 | -0.334 | -0.6048 | No |
| 87 | PLK4 |  |  | 551 | -0.334 | -0.6029 | No |
| 88 | EIF4EBP2 |  |  | 552 | -0.335 | -0.6010 | No |
| 89 | HAUS6 |  |  | 555 | -0.337 | -0.6023 | No |
| 90 | ZNF90 |  |  | 557 | -0.338 | -0.6020 | No |
| 91 | MCM3 |  |  | 560 | -0.340 | -0.6033 | No |
| 92 | HAT1 |  |  | 561 | -0.340 | -0.6014 | No |
| 93 | SASS6 |  |  | 567 | -0.342 | -0.6075 | No |
| 94 | LSM5 |  |  | 568 | -0.342 | -0.6056 | No |
| 95 | TCF19 |  |  | 569 | -0.342 | -0.6036 | No |
| 96 | CKLF |  |  | 574 | -0.347 | -0.6081 | No |
| 97 | CCDC15 |  |  | 576 | -0.347 | -0.6078 | No |
| 98 | HMGB3 |  |  | 577 | -0.348 | -0.6058 | No |
| 99 | RCC1 |  |  | 582 | -0.350 | -0.6103 | No |
| 100 | GEN1 |  |  | 584 | -0.351 | -0.6099 | No |
| 101 | DDX39A |  |  | 590 | -0.353 | -0.6159 | No |
| 102 | CDK5RAP2 |  |  | 591 | -0.353 | -0.6139 | No |
| 103 | HSPB11 |  |  | 594 | -0.354 | -0.6152 | No |
| 104 | CENPC |  |  | 596 | -0.356 | -0.6148 | No |
| 105 | RNASEH2B |  |  | 599 | -0.356 | -0.6160 | No |
| 106 | HIRIP3 |  |  | 603 | -0.359 | -0.6188 | No |
| 107 | ZWILCH |  |  | 604 | -0.360 | -0.6167 | No |
| 108 | PRIM2 |  |  | 607 | -0.362 | -0.6179 | No |
| 109 | PARP2 |  |  | 609 | -0.362 | -0.6175 | No |
| 110 | WDHD1 |  |  | 610 | -0.363 | -0.6154 | No |
| 111 | PRTFDC1 |  |  | 613 | -0.364 | -0.6166 | No |
| 112 | TOPBP1 |  |  | 614 | -0.364 | -0.6145 | No |
| 113 | BTG3 |  |  | 615 | -0.365 | -0.6125 | No |
| 114 | RFWD3 |  |  | 617 | -0.365 | -0.6120 | No |
| 115 | ZGRF1 |  |  | 620 | -0.367 | -0.6132 | No |
| 116 | HNRNPF |  |  | 621 | -0.369 | -0.6111 | No |
| 117 | DDX11 |  |  | 622 | -0.370 | -0.6090 | No |
| 118 | WDR76 |  |  | 626 | -0.373 | -0.6117 | No |
| 119 | EXOSC8 |  |  | 630 | -0.376 | -0.6144 | No |
| 120 | RNASEH2A |  |  | 633 | -0.378 | -0.6155 | No |
| 121 | RMI1 |  |  | 637 | -0.381 | -0.6182 | No |
| 122 | MPHOSPH9 |  |  | 644 | -0.384 | -0.6257 | Yes |
| 123 | PID1 |  |  | 645 | -0.385 | -0.6235 | Yes |
| 124 | TTF2 |  |  | 646 | -0.386 | -0.6213 | Yes |
| 125 | ZNF704 |  |  | 649 | -0.392 | -0.6223 | Yes |
| 126 | POLE |  |  | 652 | -0.395 | -0.6233 | Yes |
| 127 | TMEM106C |  |  | 654 | -0.397 | -0.6226 | Yes |
| 128 | SHCBP1 |  |  | 655 | -0.398 | -0.6204 | Yes |
| 129 | GINS2 |  |  | 658 | -0.399 | -0.6214 | Yes |
| 130 | SEPHS1 |  |  | 661 | -0.401 | -0.6223 | Yes |
| 131 | PBX3 |  |  | 662 | -0.403 | -0.6200 | Yes |
| 132 | MNS1 |  |  | 666 | -0.408 | -0.6225 | Yes |
| 133 | SAE1 |  |  | 668 | -0.408 | -0.6218 | Yes |
| 134 | DSN1 |  |  | 671 | -0.410 | -0.6227 | Yes |
| 135 | KIF18A |  |  | 673 | -0.412 | -0.6220 | Yes |
| 136 | NUDT1 |  |  | 674 | -0.413 | -0.6197 | Yes |
| 137 | NUP50 |  |  | 677 | -0.415 | -0.6206 | Yes |
| 138 | CIT |  |  | 680 | -0.418 | -0.6214 | Yes |
| 139 | RBMX |  |  | 682 | -0.421 | -0.6207 | Yes |
| 140 | H2AFV |  |  | 684 | -0.423 | -0.6199 | Yes |
| 141 | CENPO |  |  | 686 | -0.424 | -0.6191 | Yes |
| 142 | RPA2 |  |  | 688 | -0.424 | -0.6183 | Yes |
| 143 | CENPQ |  |  | 689 | -0.425 | -0.6159 | Yes |
| 144 | FANCA |  |  | 691 | -0.425 | -0.6151 | Yes |
| 145 | STIL |  |  | 692 | -0.425 | -0.6127 | Yes |
| 146 | CKAP5 |  |  | 694 | -0.426 | -0.6119 | Yes |
| 147 | SLC25A5 |  |  | 695 | -0.426 | -0.6095 | Yes |
| 148 | DIAPH3 |  |  | 696 | -0.427 | -0.6071 | Yes |
| 149 | RANBP1 |  |  | 697 | -0.427 | -0.6047 | Yes |
| 150 | ANP32B |  |  | 699 | -0.431 | -0.6038 | Yes |
| 151 | E2F3 |  |  | 700 | -0.431 | -0.6014 | Yes |
| 152 | CENPL |  |  | 703 | -0.432 | -0.6022 | Yes |
| 153 | CHEK1 |  |  | 704 | -0.433 | -0.5997 | Yes |
| 154 | PARPBP |  |  | 705 | -0.434 | -0.5973 | Yes |
| 155 | TIMELESS |  |  | 707 | -0.435 | -0.5964 | Yes |
| 156 | TEX30 |  |  | 709 | -0.436 | -0.5956 | Yes |
| 157 | POLD3 |  |  | 710 | -0.437 | -0.5931 | Yes |
| 158 | ZNF93 |  |  | 712 | -0.438 | -0.5922 | Yes |
| 159 | LMNB2 |  |  | 713 | -0.438 | -0.5897 | Yes |
| 160 | RBBP8 |  |  | 714 | -0.440 | -0.5873 | Yes |
| 161 | RAD18 |  |  | 715 | -0.443 | -0.5847 | Yes |
| 162 | ALYREF |  |  | 716 | -0.444 | -0.5822 | Yes |
| 163 | SLC20A1 |  |  | 717 | -0.444 | -0.5797 | Yes |
| 164 | SKA1 |  |  | 719 | -0.447 | -0.5788 | Yes |
| 165 | MASTL |  |  | 721 | -0.451 | -0.5779 | Yes |
| 166 | TTK |  |  | 724 | -0.454 | -0.5785 | Yes |
| 167 | PCNT |  |  | 725 | -0.454 | -0.5759 | Yes |
| 168 | POLQ |  |  | 726 | -0.455 | -0.5734 | Yes |
| 169 | LINC01224 |  |  | 727 | -0.455 | -0.5708 | Yes |
| 170 | RAD21 |  |  | 728 | -0.458 | -0.5682 | Yes |
| 171 | SPAG5 |  |  | 729 | -0.458 | -0.5656 | Yes |
| 172 | GINS1 |  |  | 730 | -0.459 | -0.5630 | Yes |
| 173 | CCNF |  |  | 732 | -0.460 | -0.5620 | Yes |
| 174 | POLA1 |  |  | 733 | -0.460 | -0.5594 | Yes |
| 175 | BRIP1 |  |  | 734 | -0.461 | -0.5568 | Yes |
| 176 | CSE1L |  |  | 736 | -0.461 | -0.5558 | Yes |
| 177 | C19orf48 |  |  | 738 | -0.462 | -0.5548 | Yes |
| 178 | LBR |  |  | 739 | -0.463 | -0.5522 | Yes |
| 179 | E2F8 |  |  | 741 | -0.467 | -0.5512 | Yes |
| 180 | MGME1 |  |  | 742 | -0.468 | -0.5485 | Yes |
| 181 | SMC3 |  |  | 743 | -0.470 | -0.5459 | Yes |
| 182 | NUCKS1 |  |  | 744 | -0.471 | -0.5432 | Yes |
| 183 | PTMA |  |  | 745 | -0.475 | -0.5405 | Yes |
| 184 | CHD7 |  |  | 746 | -0.480 | -0.5378 | Yes |
| 185 | TK1 |  |  | 747 | -0.480 | -0.5351 | Yes |
| 186 | DDIAS |  |  | 748 | -0.482 | -0.5324 | Yes |
| 187 | LIG1 |  |  | 752 | -0.485 | -0.5344 | Yes |
| 188 | PRR11 |  |  | 755 | -0.488 | -0.5349 | Yes |
| 189 | CDCA3 |  |  | 759 | -0.497 | -0.5369 | Yes |
| 190 | MMS22L |  |  | 761 | -0.500 | -0.5357 | Yes |
| 191 | GMNN |  |  | 766 | -0.510 | -0.5393 | Yes |
| 192 | RTKN2 |  |  | 767 | -0.511 | -0.5364 | Yes |
| 193 | LRR1 |  |  | 768 | -0.512 | -0.5335 | Yes |
| 194 | CMC2 |  |  | 771 | -0.518 | -0.5338 | Yes |
| 195 | CDC45 |  |  | 773 | -0.521 | -0.5324 | Yes |
| 196 | DBI |  |  | 776 | -0.526 | -0.5327 | Yes |
| 197 | RFC4 |  |  | 777 | -0.527 | -0.5297 | Yes |
| 198 | STMN1 |  |  | 778 | -0.529 | -0.5267 | Yes |
| 199 | DEPDC1B |  |  | 779 | -0.533 | -0.5237 | Yes |
| 200 | GLCCI1 |  |  | 780 | -0.535 | -0.5207 | Yes |
| 201 | SKA3 |  |  | 783 | -0.539 | -0.5208 | Yes |
| 202 | NCAPD2 |  |  | 784 | -0.540 | -0.5178 | Yes |
| 203 | CEP152 |  |  | 785 | -0.542 | -0.5147 | Yes |
| 204 | BLM |  |  | 786 | -0.543 | -0.5116 | Yes |
| 205 | FANCD2 |  |  | 787 | -0.544 | -0.5086 | Yes |
| 206 | RPA3 |  |  | 788 | -0.550 | -0.5054 | Yes |
| 207 | NCAPD3 |  |  | 790 | -0.559 | -0.5039 | Yes |
| 208 | ATAD5 |  |  | 791 | -0.559 | -0.5007 | Yes |
| 209 | HAUS8 |  |  | 792 | -0.560 | -0.4976 | Yes |
| 210 | HMGN2 |  |  | 795 | -0.564 | -0.4976 | Yes |
| 211 | RACGAP1 |  |  | 796 | -0.566 | -0.4944 | Yes |
| 212 | HMGB1 |  |  | 797 | -0.567 | -0.4912 | Yes |
| 213 | ASF1B |  |  | 798 | -0.568 | -0.4880 | Yes |
| 214 | CENPJ |  |  | 799 | -0.568 | -0.4848 | Yes |
| 215 | HES6 |  |  | 800 | -0.569 | -0.4815 | Yes |
| 216 | RFC3 |  |  | 801 | -0.569 | -0.4783 | Yes |
| 217 | TUBB |  |  | 802 | -0.570 | -0.4751 | Yes |
| 218 | TUBB4B |  |  | 804 | -0.573 | -0.4735 | Yes |
| 219 | CENPN |  |  | 805 | -0.574 | -0.4702 | Yes |
| 220 | USP1 |  |  | 806 | -0.575 | -0.4670 | Yes |
| 221 | CDC6 |  |  | 807 | -0.584 | -0.4637 | Yes |
| 222 | CDCA8 |  |  | 808 | -0.585 | -0.4603 | Yes |
| 223 | CHAF1A |  |  | 810 | -0.588 | -0.4586 | Yes |
| 224 | DEK |  |  | 812 | -0.591 | -0.4569 | Yes |
| 225 | FOXM1 |  |  | 814 | -0.593 | -0.4552 | Yes |
| 226 | CDCA2 |  |  | 816 | -0.597 | -0.4534 | Yes |
| 227 | MCM7 |  |  | 817 | -0.599 | -0.4500 | Yes |
| 228 | LMNB1 |  |  | 818 | -0.606 | -0.4466 | Yes |
| 229 | MCM8 |  |  | 819 | -0.607 | -0.4431 | Yes |
| 230 | MND1 |  |  | 821 | -0.615 | -0.4413 | Yes |
| 231 | CKAP2L |  |  | 826 | -0.625 | -0.4442 | Yes |
| 232 | CCDC34 |  |  | 827 | -0.626 | -0.4406 | Yes |
| 233 | DNAJC9 |  |  | 828 | -0.633 | -0.4370 | Yes |
| 234 | AURKB |  |  | 829 | -0.634 | -0.4335 | Yes |
| 235 | HELLS |  |  | 830 | -0.635 | -0.4299 | Yes |
| 236 | DTL |  |  | 831 | -0.637 | -0.4263 | Yes |
| 237 | PCNA |  |  | 832 | -0.640 | -0.4226 | Yes |
| 238 | BUB3 |  |  | 834 | -0.641 | -0.4206 | Yes |
| 239 | CENPW |  |  | 835 | -0.643 | -0.4170 | Yes |
| 240 | ITGB3BP |  |  | 838 | -0.661 | -0.4165 | Yes |
| 241 | E2F7 |  |  | 840 | -0.675 | -0.4143 | Yes |
| 242 | ANP32E |  |  | 841 | -0.676 | -0.4104 | Yes |
| 243 | KIF22 |  |  | 842 | -0.678 | -0.4066 | Yes |
| 244 | BIRC5 |  |  | 843 | -0.679 | -0.4028 | Yes |
| 245 | CENPM |  |  | 844 | -0.682 | -0.3989 | Yes |
| 246 | NCAPH |  |  | 845 | -0.685 | -0.3950 | Yes |
| 247 | BRCA1 |  |  | 846 | -0.691 | -0.3911 | Yes |
| 248 | ZWINT |  |  | 847 | -0.692 | -0.3872 | Yes |
| 249 | SPC24 |  |  | 848 | -0.694 | -0.3833 | Yes |
| 250 | BRCA2 |  |  | 850 | -0.694 | -0.3810 | Yes |
| 251 | DHFR |  |  | 851 | -0.701 | -0.3770 | Yes |
| 252 | VRK1 |  |  | 852 | -0.711 | -0.3730 | Yes |
| 253 | KIF2C |  |  | 855 | -0.721 | -0.3721 | Yes |
| 254 | NUF2 |  |  | 856 | -0.721 | -0.3680 | Yes |
| 255 | CENPK |  |  | 857 | -0.722 | -0.3640 | Yes |
| 256 | FAM111A |  |  | 858 | -0.727 | -0.3598 | Yes |
| 257 | SYNE2 |  |  | 859 | -0.728 | -0.3557 | Yes |
| 258 | ANLN |  |  | 860 | -0.733 | -0.3516 | Yes |
| 259 | KIF4A |  |  | 861 | -0.736 | -0.3474 | Yes |
| 260 | FEN1 |  |  | 862 | -0.736 | -0.3433 | Yes |
| 261 | FBXO5 |  |  | 864 | -0.742 | -0.3407 | Yes |
| 262 | CCNB2 |  |  | 865 | -0.745 | -0.3365 | Yes |
| 263 | RRM1 |  |  | 866 | -0.749 | -0.3322 | Yes |
| 264 | ARHGAP11A |  |  | 867 | -0.756 | -0.3279 | Yes |
| 265 | NDC80 |  |  | 869 | -0.762 | -0.3252 | Yes |
| 266 | ORC6 |  |  | 870 | -0.766 | -0.3209 | Yes |
| 267 | BUB1B |  |  | 871 | -0.766 | -0.3166 | Yes |
| 268 | KIF23 |  |  | 873 | -0.772 | -0.3138 | Yes |
| 269 | MELK |  |  | 874 | -0.776 | -0.3094 | Yes |
| 270 | TMPO |  |  | 875 | -0.778 | -0.3050 | Yes |
| 271 | KIF20B |  |  | 876 | -0.779 | -0.3006 | Yes |
| 272 | CKAP2 |  |  | 878 | -0.781 | -0.2978 | Yes |
| 273 | NCAPG2 |  |  | 879 | -0.781 | -0.2934 | Yes |
| 274 | PTTG1 |  |  | 880 | -0.791 | -0.2889 | Yes |
| 275 | MCM10 |  |  | 881 | -0.798 | -0.2844 | Yes |
| 276 | H2AFX |  |  | 882 | -0.799 | -0.2799 | Yes |
| 277 | KIF14 |  |  | 885 | -0.809 | -0.2785 | Yes |
| 278 | EZH2 |  |  | 886 | -0.810 | -0.2739 | Yes |
| 279 | FAM111B |  |  | 888 | -0.822 | -0.2709 | Yes |
| 280 | KIF15 |  |  | 890 | -0.834 | -0.2678 | Yes |
| 281 | CDCA5 |  |  | 892 | -0.841 | -0.2646 | Yes |
| 282 | H2AFZ |  |  | 893 | -0.849 | -0.2598 | Yes |
| 283 | RAD51AP1 |  |  | 894 | -0.853 | -0.2550 | Yes |
| 284 | DBF4 |  |  | 896 | -0.864 | -0.2517 | Yes |
| 285 | SMC2 |  |  | 897 | -0.867 | -0.2468 | Yes |
| 286 | CENPE |  |  | 899 | -0.873 | -0.2435 | Yes |
| 287 | PBK |  |  | 900 | -0.880 | -0.2385 | Yes |
| 288 | FANCI |  |  | 901 | -0.882 | -0.2335 | Yes |
| 289 | SMC4 |  |  | 902 | -0.886 | -0.2285 | Yes |
| 290 | DUT |  |  | 903 | -0.887 | -0.2235 | Yes |
| 291 | MIS18BP1 |  |  | 904 | -0.889 | -0.2185 | Yes |
| 292 | C21orf58 |  |  | 905 | -0.893 | -0.2134 | Yes |
| 293 | CCNB1 |  |  | 906 | -0.895 | -0.2084 | Yes |
| 294 | CCNE2 |  |  | 907 | -0.895 | -0.2033 | Yes |
| 295 | XRCC2 |  |  | 908 | -0.907 | -0.1982 | Yes |
| 296 | CKS1B |  |  | 909 | -0.912 | -0.1930 | Yes |
| 297 | HJURP |  |  | 910 | -0.921 | -0.1878 | Yes |
| 298 | CKS2 |  |  | 911 | -0.922 | -0.1826 | Yes |
| 299 | GTSE1 |  |  | 912 | -0.941 | -0.1772 | Yes |
| 300 | KIF11 |  |  | 913 | -0.957 | -0.1718 | Yes |
| 301 | MYBL2 |  |  | 914 | -0.959 | -0.1664 | Yes |
| 302 | CLSPN |  |  | 916 | -0.964 | -0.1626 | Yes |
| 303 | TYMS |  |  | 917 | -0.965 | -0.1571 | Yes |
| 304 | CENPU |  |  | 918 | -0.971 | -0.1516 | Yes |
| 305 | PRC1 |  |  | 919 | -0.980 | -0.1461 | Yes |
| 306 | MAD2L1 |  |  | 921 | -0.990 | -0.1421 | Yes |
| 307 | UBE2T |  |  | 922 | -0.994 | -0.1365 | Yes |
| 308 | SPC25 |  |  | 924 | -1.033 | -0.1322 | Yes |
| 309 | HIST1H1E |  |  | 925 | -1.049 | -0.1263 | Yes |
| 310 | ESCO2 |  |  | 926 | -1.067 | -0.1202 | Yes |
| 311 | UBE2C |  |  | 928 | -1.108 | -0.1156 | Yes |
| 312 | NUSAP1 |  |  | 929 | -1.112 | -0.1093 | Yes |
| 313 | TPX2 |  |  | 930 | -1.124 | -0.1029 | Yes |
| 314 | NCAPG |  |  | 931 | -1.142 | -0.0965 | Yes |
| 315 | ASPM |  |  | 932 | -1.146 | -0.0900 | Yes |
| 316 | ATAD2 |  |  | 933 | -1.162 | -0.0834 | Yes |
| 317 | CENPF |  |  | 935 | -1.200 | -0.0782 | Yes |
| 318 | HIST1H1C |  |  | 936 | -1.220 | -0.0713 | Yes |
| 319 | HMGB2 |  |  | 937 | -1.238 | -0.0643 | Yes |
| 320 | CDK1 |  |  | 938 | -1.248 | -0.0573 | Yes |
| 321 | RRM2 |  |  | 940 | -1.403 | -0.0510 | Yes |
| 322 | MKI67 |  |  | 941 | -1.407 | -0.0430 | Yes |
| 323 | TOP2A |  |  | 942 | -1.417 | -0.0350 | Yes |
| 324 | HIST1H1D |  |  | 944 | -1.810 | -0.0263 | Yes |
| 325 | HIST1H1B |  |  | 945 | -1.903 | -0.0156 | Yes |
| 326 | HIST1H4C |  |  | 946 | -2.752 | -0.0000 | Yes |
Table: GSEA details [plain text format]

  

Fig 2: RICHARDS\_NOWAKOWSKI\_SCIENCE\_2017\_MGE\_DIV\_UPREG: Random ES distribution      
 Gene set null distribution of ES for **RICHARDS\_NOWAKOWSKI\_SCIENCE\_2017\_MGE\_DIV\_UPREG**
